# Predictors of Academic Resilience Based on Demographic and Psychological Profiles among Undergraduate Nursing Students: A Multicentric Survey in India

**DOI:** 10.17533/udea.iee.v44n1e03

**Published:** 2026-03-28

**Authors:** Srinivasan Chelladurai, Sharanabasappa S, Vasanth Chellamuthu, Vinayagamoorthy Venogopal, Vasantha C Kalyani

**Affiliations:** 1 Nursing Faculty. Email: Sri.jipmer2018@gmail.com. Corresponding author https://orcid.org/0000-0002-7281-7197 All India Institute Of Medical Sciences India Sri.jipmer2018@gmail.com; 2 Nursing Faculty. Email: sharanu.s.d@gmail.com https://orcid.org/0000-0003-3634-721X All India Institute Of Medical Sciences India sharanu.s.d@gmail.com; 3 Nursing Faculty. Email: vcfaithmother@gmail.com https://orcid.org/0000-0002-3444-2646 All India Institute Of Medical Sciences India vcfaithmother@gmail.com; 4 Asociate professor, Department of Community and Family Medicine. Email: drvinayagamoorthy@gmail.com https://orcid.org/0000-0002-7951-8552 All India Institute Of Medical Sciences Department of Community and Family Medicine India drvinayagamoorthy@gmail.com; 5 Professor Cum Principal. Email: vasantharaj2003@gmail.com https://orcid.org/0000-0003-1075-9208 All India Institute Of Medical Sciences India vasantharaj2003@gmail.com; 6 All India Institute of Medical Science (AIIMS), Deoghar. India. All India Institute Of Medical Sciences All India Institute of Medical Science (AIIMS) Deoghar India

**Keywords:** academic resilience, psychological wellbeing, coping mechanisms, students nursing., resiliencia académica, bienestar psicológico, mecanismos de afrontamiento, estudiantes de enfermería., resiliência acadêmica, bem-estar psicológico, mecanismos de enfrentamento, estudantes de enfermagem.

## Abstract

**Objective.:**

To predict academic resilience based on demographic and psychological factors among undergraduate nursing students in India.

**Methods.:**

Cross sectional survey was conducted for 759 nursing undergraduates. We chose four nursing institutions from various regions of India to serve as the sample unit. Data was collected virtually via a self-administered questionnaire using the Bharathiar University Resilience Scale (BURS), a coping inventory, and a psychological well-being questionnaire. Log-binomial regression was used to predict academic resilience, and independent student t-tests and Pearson correlation coefficients were used to compare resilience scores and the relationship between independent variables.

**Results.:**

The primary demographic factors linked to greater academic resilience were, male undergraduates, between the ages of 24 to 30 years, enrolled in private institutions with family income of <10000 INR (1 US dollar = 87.98 INR) and institutes located in north India. Psychological well-being and academic resilience were significantly positively correlated, while cognitive coping strategies and academic resilience were significantly negatively correlated. Regression analysis demonstrates that higher psychological well-being score increases the likelihood of achieving high academic resilience. In a similar vein, they might experience a decrease in academic resilience if they were adopting a cognitive coping style.

**Conclusion.:**

The study’s findings revealed that sociodemographic and psychological variables can be predictors of academic resilience in undergraduate nursing students from India. These findings can help universities to develop target strategies to promote students’ resilience and reduce the risk of poor mental health among this population.

## Resumen

**Objetivo.:**

Predecir la resiliencia académica basándose en factores demográficos y psicológicos entre estudiantes universitarios de enfermería en la India.

**Métodos.:**

Se realizó una encuesta transversal a 759 estudiantes universitarios de enfermería de cuatro instituciones de diversas regiones de la India. Los datos se recopilaron de forma virtual mediante un cuestionario autoadministrado que utilizaba la Escala de Resiliencia de la Universidad de Bharathiar (BURS), un inventario de afrontamiento y un cuestionario de bienestar psicológico. Se utilizó una regresión log-binomial para predecir la resiliencia académica**.**

**Resultados.:**

. Las características principales de los factores demográficos relacionados con una mayor resiliencia académica fueron: estudiantes varones, entre 24 y 30 años, matriculados en instituciones privadas con ingresos familiares inferiores a 10 000 INR (1 dólar estadounidense = 87.98 INR) e institutos ubicados en el norte de la India. El bienestar psicológico y la resiliencia académica mostraron una correlación positiva significativa, mientras que las estrategias cognitivas de afrontamiento y la resiliencia académica mostraron una correlación negativa significativa. El análisis de regresión mostró que una puntuación más alta en bienestar psicológico aumenta la probabilidad de alcanzar una alta resiliencia académica. Del mismo modo, podrían experimentar una disminución de la resiliencia académica si adoptaran un estilo de afrontamiento cognitivo**.**

**Conclusión.:**

Las variables sociodemográficas y psicológicas pueden ser predictores de la resiliencia académica en los estudiantes de enfermería de la India. Estos resultados pueden ayudar a las universidades a desarrollar estrategias específicas para promover la resiliencia de los estudiantes y reducir el riesgo de mala salud mental entre esta población.

## Resumo

**Objetivo.:**

Prever a resiliência acadêmica com base em fatores demográficos e psicológicos entre estudantes universitários de enfermagem na Índia.

**Métodos.:**

Foi realizada uma pesquisa transversal com 759 estudantes universitários de enfermagem de quatro instituições de diversas regiões da Índia. Os dados foram coletados virtualmente por meio de um questionário autoaplicável que utilizava a Escala de Resiliência da Universidade de Bharathiar (BURS), um inventário de enfrentamento e um questionário de bem-estar psicológico. Utilizou-se uma regressão log-binomial para prever a resiliência acadêmica**.**

**Resultados.:**

As principais características dos fatores demográficos relacionados a uma maior resiliência acadêmica foram: estudantes do sexo masculino, entre 24 e 30 anos, matriculados em instituições privadas com renda familiar inferior a 10 000 INR (1 dólar americano = 87.98 INR) e institutos localizados no norte da Índia. O bem-estar psicológico e a resiliência acadêmica mostraram uma correlação positiva significativa, enquanto as estratégias cognitivas de enfrentamento e a resiliência acadêmica mostraram uma correlação negativa significativa. A análise de regressão mostrou que uma pontuação mais alta em bem-estar psicológico aumenta a probabilidade de alcançar uma alta resiliência acadêmica. Da mesma forma, eles poderiam experimentar uma diminuição da resiliência acadêmica se adotassem um estilo de enfrentamento cognitivo**.**

**Conclusão.:**

As variáveis sociodemográficas e psicológicas podem ser preditoras da resiliência acadêmica em estudantes de enfermagem na Índia. Esses resultados podem ajudar as universidades a desenvolver estratégias específicas para promover a resiliência dos estudantes e reduzir o risco de saúde mental precária nessa população.

## Introduction

The psychological problems of undergraduates are acknowledged as a critical public health concern.^1^ Students around 1the world report that studying nursing is stressful. Compared with students in other undergraduates, nursing students often experience great pressure from unfamiliar clinical environments, the gap between professional theory and practice, unexpected emergencies, strained relationships with patients and their families, exposure to infectious diseases, and heavy workloads.^2^ The high-stress levels experienced by nursing students can negatively affect their overall well-being and academic performance.^3^ It is widely acknowledged that resilience is essential to positive health.^4^ Researchers have examined several forms of resilience, such as behavioural, emotional, and academic resilience. Academic resilience has received particular attention lately. Academic resilience, according to Wang and Gordon, is the growing probability of academic success despite environmental obstacles and challenges brought on by novel situations and experiences.^5^ Research finding also say that, Students who possess resilience can perform well and sustain high academic achievement even in the face of stress or the potential of failure.^6^ Therefore, academic resilience is crucial for Undergraduate students. It is important to remember that resilience can change over time and show up more or less in various contexts.^7^ Numerous elements, including social, economic, and psychological elements as well as individual traits like gender and relationships, have been connected to greater resilience.^8^

Psychological well-being is a sense of feeling healthy, leading to complete awareness of personal integrity and the spiritual element of life. Additionally, studies have shown that resilience can effectively improve a person's psychological health.^9^ A healthcare student's psychological health is a crucial component of their personality for both professional and personal satisfaction.^10^ Individuals utilize a variety of coping mechanisms in stressful circumstances. The goal of effective coping is to mitigate the potential adverse effects of going through uncomfortable emotional states. Furthermore, even when confronted with the same kind of stress, people employ various coping mechanisms, which may result more from personal preferences than behavioural variations.^11^ Although the relationship between coping styles and psychological well-being and resilience has been identified, there is still scanty research on the relationship between psychological well-being, coping styles, and academic resilience. Thus, the present study aims to examine the relationship between psychological well-being and coping styles, on the one hand, and academic resilience, on the other hand. In addition, it seeks to determine the contribution of psychological well-being, coping styles and demographic characters to academic resilience. This study provides fundamental data to prepare students' intervention strategies for enhancing academic resilience by investigating its influencing factors.

## Methods

The study adopted a descriptive cross-sectional design, utilizing a self-administered questionnaire survey circulated through Google form among undergraduate nursing students at four different institutes in India. The data collection was done during December and June 2024. The inclusion criteria involved current full-time nursing students who provided informed consent and willingly participated in the study. Exclusion criteria were applied to individuals who requested withdrawal from the study. The sample size for the present study was calculated using a 5 % marginal error, 95% confidence interval, and 50% response distribution. Using these parameters, the estimated sample size comes to 368. Given a non-response of 10 %, the sample size came to 410. A total of 784 questionnaires were collected, and following the exclusion of incomplete responses, 759 valid responses were obtained, resulting in a valid re

sponse rate of 96.81%.

Measures. (i) The Bharathiar University Resilience Scale (BURS):^12^ The scale consisted of 30 items, each with a 5-point rating option, ranging from 1 (not at all appropriate) to 5 (most appropriate). The scale was designed to measure resilience across various dimensions. These dimensions include the duration it takes to return to normalcy, the reaction to negative events, the response to risk factors in academic. This resilience scale has a high level of internal consistency (The alpha is 0.94); (ii) Psychological well-being (PSW) - 20 Point scale^:(^^13^ This is a 20-item self-report measure that is composed of four factor-based subscales that measure self-acceptance (SA), mastery and competence (MC), positive relations (PR), and sense of engagement and growth (EG). All four subscales have satisfactory reliability and validity. A 6-point Likert scale, ranging from 1 (strongly disagree) to 6 (strongly agree), is used to rate each of the 20 items. The range of the total PWB raw score is 20 to 120. Therefore, a higher PWB is generally indicated by higher scores. It is possible to compute the four subscale scores as well as the overall scores; and, (iii) Coping scale:^14^^,^^15^ Participants' cognitive, emotional, and behavioral approaches to dealing with challenges and issues will be evaluated using a 13-item coping strategies questionnaire. The Coping Strategies Scale of Holahan served as the model for the cognitive and emotional approaches (items 2, 3, and 4). Hamby *et al* have adapted additional items (items 1, 5, 6, and 8) that focus on emotional and cognitive approaches. 

Data collection Procedure. An online survey was used to collect data for this investigation. Before the commencement of the study, the researchers contacted the Principals of the respective institutes to provide them with a comprehensive explanation of the research’s purpose and procedures. Following that, link to the online survey was shared among the students by their respective principals. Three strategies were used to find participating students: 1. We advertised the questionnaire on social media, like WhatsApp group and official email. 2. We printed the link and put it up in departments, dining areas, and classrooms so students could scan it and fill it out. 3. We encouraged students to share the link to their peers.The participants answered the questions through the survey link. Before the survey, they were informed of the study's goal and the voluntary nature of participation. They were told that they could end the study at any moment and that answering the questions on the questionnaire signified that they gave their informed consent to take part in the research. All the information was gathered voluntarily, anonymously, and confidently. This study was approved by the Institution Ethics Committee.

Data analysis. Descriptive statistics, such as frequencies and percentages for categorical variables and mean and Standard deviation (SD) for non-normally distributed continuous variables, were used to analyze the demographic data. The log-binomial regression analysis explored the variables influencing academic resilience among nursing students. Crude and adjusted prevalence ratio was calculated and reported with its 95% Confidence Interval. A statistically significant difference was defined as *p*<0.05. The Stata software version 12.0 was used for the analyses. 

Ethical issues: The Institutional Ethics Committee of All India Institute of Medical Science (AIIMS), Deoghar. India, approved this study. (IEC CODE: 2023-96-IND-03).

## Results

### Participant’s characteristics

A total of 759 nursing students participated in the study, with a mean age of 20.6 ± 1.8 years. The majority of participants (64.7%) were in the 20-23 years age group and most were female (82.0%). More than one-third (35.2%) were in their second year of study. Most students were from the northern region of India (45.9%) and were enrolled in private institutions (69.8%). Half of the students (50.7%) were residing at home. According to the International Physical Activity Questionnaire (IPAQ), over half (53.1%) reported moderate physical activity, and no participants engaged in heavy physical activity. The majority (73.9%) used the internet for less than four hours per day, primarily for academic and social purposes (72.5%). (Table 1)

**Table 1:** Social-Demographical distribution of study participants (*n*=759)

Character	n (%)
Age; mean (SD)	20.6 (1.8)
Age group; *n* (%)	
17 - 19	215 (28.33)
20 - 23	491 (64.69)
24 - 30	53 (6.98)
Gender; *n* (%)	
Male	136 (17.92)
Female	623 (82.0)
Year of the course; *n* (%)	
I	231 (30.43)
II	267 (35.18)
III	205 (27.01)
IV	56 (7.38)
Ethnicity; *n* (%)	
South	92 (12.12)
North	348 (45.85)
West	263 (34.65)
East	56 (7.38)
Type of Organization; *n* (%)	
Private	530 (69.83)
Deemed	25 (3.29)
Government	204 (26.88)
Residence; *n* (%)	
Hostel	279 (36.76)
Home	385 (50.72)
Other	95 (12.52)
Total monthly Family Income;* *n* (%)	
>10000	243 (32.02)
10000-250000	216 (28.46)
25000-50000	159 (20.95)
<500000	141 (18.58)
Duration of Internet use per day; *n* (%)	
>4 hours	561 (73.91)
<4 hours	198 (26.09)
Purpose of Internet use; *n* (%)	
Academic	61 (8.04)
Social	64 (8.43)
Academic & Social	550 (72.46)
Other	84 (11.07)
Level of Physical Activity; *n* (%)	
Moderate	403 (53.10)
Sedentary	356 (46.90)
Resilience; *n* (%)	
Low	490 (64.56)
High	269 (35.44)
Resilience; mean (SD)	86.26 (15.9)
Psychological well-being;mean (SD)	
Self-acceptance	8.28 (3.62)
Mastery & Competence	25.84 (6.76)
Positive relation	18.04 (3.96)
Engagement & Growth	9.05 (4.23)
Total well-being	61.23 (10.25)
Copingmean (SD)	
Emotional	18.24 (3.24)
Cognitive	21.78 (3.68)

*: 1 US dollar = 87.98 INR

Scores of the main variables

The majority of participants had 490 (64.6%) low academic resilience, while the mean (SD) academic resilience score was 86.26 (15.9), and the mean (SD) score of psychological well-being was 61.23 (10.25). The mean (SD) sub-scale measurement of psychological well-being scores, namely, self-acceptance, mastery & competence, positive relation, and engagement & growth, were 8.28 (3.26), 25.84 (6.76), 18.04 (3.96), and 9.05 (4.23) respectively. The mean (SD) score of emotional scoping was 18.24 (3.24), and cognitive coping was 21.78 (3.68). (Table 1) Comparing the mean (SD) score of various sub-scales of psychological well-being and coping strategies among students with low and high academic resilience, positive relation and engagement & growth had significantly higher mean scores among students with high academic resilience. The total mean score of the psychological well-being scale was also statistically significantly higher among those students with higher academic resilience. There was no difference in the mean score of the coping strategy scale between low and high academic resilience students. (Table 2) There was a statistically significant, good positive correlation (r=0.68) between engagement & growth and self-acceptance, and similarly, between the emotional and cognitive aspects (r=0.62). The correlation between various subscales of psychological well-being and coping strategies is shown in Table 3.

**Table 2:** Association between study variable and academic resilience

Variables	Resilience			***p*-value**
	Low (Mean/SD)	High (Mean/SD)	
Psychological well-being			
Self-acceptance	08.0 (3.5)	8.75 (3.7)	0.70
Mastery & Competence	25.8 (7.0)	25.8 (6.3)	0.053
Positive relation	17.6 (4.2)	18.7 (3.4)	0.002
Engagement & Growth	08.8 (3.9)	09.4 (4.6)	0.004
Total	60.3 (10.6)	62.0 (9.3)	0.009
Coping strategies			
Cognitive aspects	21.98 (3.7)	21.42 (3.7)	0.76
Emotional aspects	18.35 (3.4)	18.03 (3.4)	0.92

**Table 3:** Correlations of the studied variables

Variables	1	2	3	4	5	6
Self-acceptance (1)						
Mastery & Competence (2)	-0.32**					
Positive relation (3)	0.01*	0.36**				
Engagement & growth (4)	0.68**	-0.26**	0.09*			
Cognitive aspects (5)	-0.40**	0.29**	0.02	-0.36**		
Emotional aspects (6)	-0.36**	0.30**	0.07*	-0.33**	0.62**	
Resilience	0.08*	0.03	0.19**	0.06*	-0.08*	-0.03

*p<0.05, ** *p*<0.001

**Table 4:** Predictors of academic resilience among Nursing students

Characteristics	Resilience		CPR (95 % Cl)		***p*-value** ^#^	APR (95% CI)		***p*-value** ^#^
	Low n (%)^		High n (%)^
Age in years
17-19	164 (76.2)		51 (23.7)	Ref (1)		-	Ref (1)	-
20-23	298 (60.6)		193 (39.3)	1.6 (1.3 - 2.1)		<0.001	1.5 (1.2 - 2.0)	<0.001
24-30	28 (52.8)		25 (47.1)	1.9 (1.4 - 2.9)		<0.001	1.3 (0.9 - 1.8)	0.08
Gender
Male	71 (52.2)		65 (47.7)	1.4 (1.2 - 1.7)		<0.001	0.7 (0.6 -0.9)	0.01
Female	419 (67.2)		204 (32.7)	Ref (1)		-	Ref (1)	-
Year of the course
I year	172 (74.4)		59 (25.5)	4.8 (1.5 -14.6)		0.006	4.9 (1.8 -13.1)	<0.001
II year	181 (67.7)		86 (32.2)	6.0 (1.9 - 18.3)		0.002	5.8 (2.2 -15.3)	<0.001
III year	84 (40.9)		121 (59.0)	11.0 (3.6 - 33.3)		<0.001	8.3 (3.1 - 21.7)	<0.001
IV year	53 (94.6)		3 (5.3)	Ref (1)		-	Ref (1)	-
Ethnicity
South	86 (93.4)		6 (6.5)	Ref (1)		-	Ref (1)	-
North	258 (74.1)		90 (25.8)	3.9 (1.7 - 8.7)		0.001	2.6 (1.3 - 5.5)	0.008
West	103 (39.1)		160 (60.8)	9.3 (4.2 - 20.3)		<0.001	5.2 (2.5 - 10.7)	<0.001
East	43 (76.7)		13 (23.2)	3.5 (1.4 - 8.8)		0.006	2.03 (0.8 - 4.7)	0.10
Type of Organization
Private	270 (50.9)		260 (49)	12.5 (6.3 -24.8)		<0.001	10.2 (1.3 - 15.5)	0.008
Deemed	24 (69)		1 (4)	1.0 (0.1 - 7.8)		0.985	5.2 (2.5 - 10.7)	<0.001
Government	196 (96.0)		8 (3.9)	Ref (1)		-	Ref (1)	-
Place of stay
Hostel	222 (79.5)		57 (20.4)	Ref (1)		-	Ref (1)	-
Home	242 (62.8)		143 (37.1)	1.8 (1.3 - 2.3)		<0.001	1.0 (0.8 - 1.2)	0.89
Other	26 (27.3)		69 (72.6)	3.5 (2.7 - 4.6)		<0.001	1.5 (1.2 - 1.9)	<0.001
Total Monthly Family Income
<10000	113 (46.5)		130 (53.5)	1.8 (1.4 - 2.5)		<0.001	1.5 (1.2 - 1.8)	<0.001
10001-25000	154 (71.3)		62 (28.7)	1.0 (0.7 - 1.4)		0.94	1.0 (0.8 - 1.3)	0.75
25001-50000	122 (76.7)		37 (23.2)	0.8 (0.5 - 1.2)		0.31	0.9 (0.7 - 1.3)	0.92
<50000	101 (71.6)		40(28.3)	Ref (1)		-	Ref (1)	-
Duration of Internet use per day
>4 hours	357 (63.6)		204 (36.3)	1.1 (0.8 - 1.4)		0.37	NA
<4 hours	133 (67.1)		65 (32.8)	Ref (1)		-
Purpose of Internet use
Academic	43 (70.4)		18 (29.5)	Ref (1)			NA
Social	44 (68.7)		20 (31.2)	1.0 (0.6 - 1.8)		0.83
Both	347 (63.0)		203 (36.9)	1.2 (0. 8 - 1.8)		0.26
Other	56 (66.6)		28 (33.3)	1.12 (0.7 - 1.8)		0.67
Level of Physical Activity
Moderate	254 (63)		149 (36.9)	1.09 (0.9 - 1.3)		0.34	NA
Sedentary	236 (66.3)		120 (33.7)	Ref (1)		-
Psychological wellbeing and coping style
Psychological wellbeing	NA		1.04 (1.0 - 1.1)		0.002	1.2 (1.01 - 1.6)		0.04
Emotional coping	NA		1.0 (0.9 - 1.2)		0.208	NA	
Cognitive coping	NA		0.9 (0.9 -1.0)		0.04	1.0 (0.9 -1.0)		0.340
*Note: ^Row %, CPR-Crude Prevalence Ratio, APR-Adjusted Prevalence Ratio, CI-Confidence Interval, Ref-Reference category, # depends on log-binomial regression*

The prevalence of high academic resilience among participants aged 24 - 30 was 47.1% compared to that of 23.7% among those aged 17 - 19. Thus, the crude prevalence of high resilience rate was 1.9 times (95% CI: 1.4 - 2.9) significantly higher for those aged 24 - 30 compared to 17 - 19 years students. Compared to females, males had 1.4 times (95% CI: 1.2 - 1.7) significantly higher prevalence of resilience in the unadjusted analysis. Students belonging to I year had better resilience than the final year (CPR-4.8; 95% CI: 1.5 -14.6), students from the north, west, and east had better resilience than those from the south, compared to government organizations those from private had better resilience (CPR-12.5; 95% CI: 6.3 -24.8). Those who stayed at home had better resilience (CPR-1.8; 95% CI: 1.3 - 2.3) than those at the hostel. Those factors that were significantly associated with high academic resilience were included in the adjusted analysis and that revealed that students aged 20-23 years (APR-1.5; 95% CI: 1.2 - 2.0), females, I, II- & III-year students, and those from North & West, deemed university (APR-5.2; 95% CI: 2.5 - 10.7), stay in other places, and with a total monthly family income of <10,000 rupees (APR-1.5; 95% CI: 1.2 - 1.8) had significantly better resilience than their counterparts**.** Every unit increase in psychological well-being score there is 1.04 times chance to be in high resilience group and this was statistically significant in unadjusted analysis. Similarly, every unit increase in cognitive coping score there is 90% chance for being in low resilience group. They are negatively associated in unadjusted analysis. There was no association between emotional coping and academic resilience. In adjusted analysis also found that psychological well-being is positively associated with academic resilience**.** (Table 4)

### Discussion

Resilience could be viewed as a process for adapting to adversity and stress. Coping styles and wellbeing are internal protective factors which affect resilience. The present study is predicting the resilience based on demographic and coping, psychological well-being among 759 undergraduate nursing students from different institute across India. The study findings in each variable represent impressive result in Nursing undergraduates. Our study found that among undergraduate nursing students age group of 24 - 30 years was significantly associated with higher resilience compared to age group of 17-19 years. Gender was associated with academic resilience on log-binomial regression, which showed that males had higher resilience scores when compared to females. Previous studies also reported similar finding before and after covid-19 pandemic among undergraduate students.^16^^,^^17^ However, there was a lack of gender differences among undergraduate students in Brazil and Oman.^18^^,^^19^ Students with low family income or whose family income was < 10000 Indian rupees had 1.8 times higher resilience scores than those with higher family income or whose family income was> 10000 India rupees. These findings are consistent with a previous study conducted in Brazil, Omani students that focused on the monthly family income with resilience.^18^^,^^19^ However, these results were not supported by de Andrade *et al.*,^20^ which reported that Students with higher family income had higher resilience scores than those with lower family income. Resilience among Egyptian medical students reported Spending more than 2 hours on internet was significantly associated with low resilience. This finding was contradicted to the current study finding, Indian undergraduate nursing students didn’t show any significant association with internet usage and academic resilience.^21^

The current study shows a strong correlation between nursing students' coping mechanisms and psychological health and resilience. Nonetheless, it was discovered that greater academic resilience may result from greater wellbeing. A similar pattern was noted in the research by Sagone and Caroli.^22^ who found that, apart from life purpose, greater resilience is directly linked to improved psychological well-being. Resilience and psychological well-being among medical students did not correlate, according to conflicting findings from a study by Sonika *et al*.^23^ Coping strategies are predictors of academic resilience, according to the study's findings. In the current study, cognitive coping style and resilience are negatively correlated. The results of this study are consistent with research by Kawata *et al*.,^24^ which found that high resilient people prefer to manage stressful situations by using problem-focused coping strategies (such as effort and activecoping) rather than emotion-focused coping strategies (such as behavioral disengagement and emotional venting). In fact, we found no difference in academic resilience scores between cognitive and emotional coping strategies. However, this finding was not supported by study conducted Campbell-Sills *et al*.^25^

There are significant ramifications for nursing education from these findings. Resilient students are more likely to exhibit better stress management, academic tenacity, and flexibility in the face of difficulties in both clinical and educational contexts. The varying correlations between psychological well-being and resilience imply that treatments that foster resilience might need to be customised based on institutional circumstances, coping strategies, and student demographics. Programs that focus on cognitive coping mechanisms, for example, may lessen susceptibility in students who are not as resilient, while encouraging participation and development may improve self-acceptance and general academic achievement. These revelations offer concrete proof that educators and legislators may put specific plans into place to boost academic performance, foster psychological well-being, and increase resilience in nursing students.

There are very few studies which measured academic resilience among Indian nursing students and explored factors related to it, such as psychological well-being, coping strategies, and other sociodemographic traits, both academic and non-academic. In addition, we have a multi-centric study design that includes universities from various regions of India in an effort to give the results reliability and generalizability. It is crucial to remember that a cross-sectional study only identifies the relationships between independent variables and resilience at a single point in time; it cannot identify causality or changes in resilience. This restriction is essential to comprehending the extent of our research. Recall bias, overestimation, and/or underestimation of the data may have resulted from the use of an online, self-reported questionnaire with past-related questions. The self-reported nature of the questionnaire introduces potential social desirability bias, even though it uses a validated measure of resilience. This is because respondents may attempt to answer in a way that they believe to be more socially desirable. By guaranteeing anonymity, we sought to reduce this bias. Students' perceptions of their academic resilience and other study variables may have been impacted by the special circumstances surrounding the post-pandemic survey, which is another possible source of response bias. When interpreting the survey's results, it is critical to take the Indian context into account. Since most research usually uses small samples of adolescents and schoolchildren, these features set our study apart from other studies carried out in India and around the world. Future longitudinal research can contribute to a deeper understanding of the risk factors and academic resilience of students.

Conclusion. A study conducted among undergraduate nursing students revealed that male nursing students, students with age group of 24-30 years, students from first year nursing in private organizations, and institutes located in the north India compared to the southern part of India-were the main demographic factors associated with higher academic resilience. Academic resilience has a significant positive correlation with psychological well-being and a negative correlation with cognitive coping strategies. In regression analysis, it shows that an increase in psychological well-being score will give a chance of getting high academic resilience. Similarly, if they were using a cognitive coping style, there would be a chance of getting lower academic resilience.

## References

[1] Lei XY, Xiao LM, Liu YN, Li YM (2016). Prevalence of depression among Chinese university students: a meta-analysis. PLoS One.

[2] Ahmed W, Mohammed B (2019). Nursing students’ stress and coping strategies during clinical training in KSA. Journal of Taibah University Medical Sciences.

[3] Spurr S, Walker K, Squires V, Redl N (2021). Examining nursing students’ wellness and resilience: an exploratory study. Nurse Education in Practice.

[4] Phillips SP, Reipas K, Zelek B (2019). Stresses, strengths and resilience in adolescents: A qualitative study. Journal of Primary Prevention.

[5] Wang MC, Gordon EW (1994). Educational resilience in inner-city America: challenges and prospects.

[6] Martin AJ, Marsh HW (2006). Academic resilience and its psychological and educational correlates: a construct validity approach. Psychology in the Schools.

[7] Southwick SM, Bonanno GA, Masten AS, Panter-Brick C, Yehuda R (2014). Resilience definitions, theory, and challenges: interdisciplinary perspectives. European Journal of Psychotraumatology.

[8] Pinheiro DPN (2004). A resiliência em discussão [Resilience in Discussion]. Psicologia em Estudo.

[9] Souri H, Hasanirad T (2011). Relationship between resilience, optimism and psychological well-being in students of medicine. Procedia - Social and Behavioral Sciences.

[10] Eley DS, Cloninger CR, Walters L, Laurence C, Synnott R, Wilkinson D (2013). The relationship between resilience and personality traits in doctors: Implications for enhancing well-being. Peer Journal of Life & Environment.

[11] Son HJ, Lee KE, Kim NS (2015). Affecting factors on academic resilience of nursing students. International Journal of u- and e-Service, Science and Technology.

[12] Annalakshimi N Research Methodology Tool and Techniques.

[13] Mehrotra S, Tripathi R, Banu H (2013). Psychological wellbeing: Reflection on an elusive construct and its assessment. The Journal of Indian Academy of Applied Psychology.

[14] Holahan CJ, Moos RH (1987). Personal and contextual determinants of coping strategies. Journal of Personality and Social Psychology.

[15] 15. Hamby S, Grych J, Banyard VL. Life Paths measurement packet: Finalized scales. Sewanee (TN): Life Paths Research Program; 2015 [cited 2025 Feb25]. Available from: Available from: http://www.lifepathsresearch.org/strengths-measures .

[16] Erdogan E, Ozdogan O, Erdogan M (2015). University Students’ Resilience Level: the effect of gender and Faculty. Procedia-Social and Behavioral Sciences.

[17] Peng L, Zhang J, Li M, Li P, Zhang Y, Zuo X (2012). Negative life events and mental health of Chinese medical students: the effect of resilience, personality and social support. Psychiatry Research.

[18] Moraes IM de, Nascimento FA do, Bastos GP, Barros S, Silva RM da, Santos ALM (2020). Fatores sociodemográficos e acadêmicos relacionados à resiliência de graduandos da área da saúde. REVISA.

[19] Al Omari O, Al Yahyaei A, Wynaden D, Damra J, Aljezawi M, Al Qaderi M (2023). Correlates of resilience among university students in Oman: a cross-sectional study. BMC Psychology.

[20] De Andrade J.E., Meireles A.L., Machado E.L. (2024). Sociodemographic, economic, and academic factors linked with resilience in university students during covid-19 pandemic: a Brazilian cross-sectional study. BMC Psychology.

[21] Mohammed HE, Bady Z, Abdelhamid ZG, Elawfi B, AboElfarh HE, Elboraay T, Abdel-Salam DM (2024). Factors influencing stress and resilience among Egyptian medical students: a multi-centric cross-sectional study. BMC Psychiatry.

[22] Sagone E, Caroli ME (2014). A correlational study on dispositional resilience, psychological well-being and coping strategies in university students. American Journal of Educational Research.

[23] Sonika Shalini, Kumar R (2019). Resilience, psychological well‑being, and coping strategies in medical students. Indian Journal of Psychiatric Nursing.

[24] 24. Kawata Y, Kamimura A, Yamada K, Izutsu S, Wakui S, Mizuno M, et al. Relationship between resilience and stress coping among Japanese university athletes [Internet]. 2015 [cited 2025 Feb 21]. Available from: Available from: https://share.google/WskfNkW4wH4Lwnt4i .

[25] Campbell Sills L, Cohan SL, Stein M (2006). Relationship of resilience to personality, coping, and psychiatric symptoms in young adults. Behaviour Research and Therapy.

